# Supplementary material for: A simple tensor network algorithm for two-dimensional steady states
Source: Nat Commun. 2017 Nov 3;8:1291. doi: 10.1038/s41467-017-01511-6 (PMC5668304; doi:10.1038/s41467-017-01511-6)
Supplement: Supplementary file 1 — Supplementary Information [file 41467_2017_1511_MOESM1_ESM.pdf]

Supplementary Note 1: Projected Entangled-Pair Operators

Projected Entangled-Pair Operators (PEPO) are simply the operator version of Projected Entangled-Pair States (PEPS), in the same way that Matrix Product Operator (MPO) are the operator version of Matrix Product States (MPS) for the $1d$ case [1–5]. More specifically, a $2d$ PEPO is an operator that acts on a $2d$ PEPS and produces a new PEPS, and admits a tensor network description as in Supplementary Fig.(1). In principle there is no restriction on the coefficients of the tensors, so that PEPOs can represent, at least a priori, operators of any kind: generic, unitary, positive, and so on. In our case we use PEPOs to describe reduced density matrices, which are positive by construction. However, a PEPO does not need to be necessarily positive, and therefore the negative eigenvalues need to be under control in order to produce an accurate representation of a physical mixed state, as explained in the main text. Moreover, one can "vectorize" the PEPO, so that the resulting object can be treated as a $2d$ PEPS with double physical indices. Importantly, in this supplementary material we add diagonal weight matrices λ at the links. This is convenient in order to implement the so-called simple update, which we comment in the next section. In practice we also used a 2-site unit cell with tensors A and B at every site, and diagonal positive matrices $\lambda_1, \lambda_2, \lambda_3$ and λ_4 at every link, as shown in Supplementary Fig.(1).

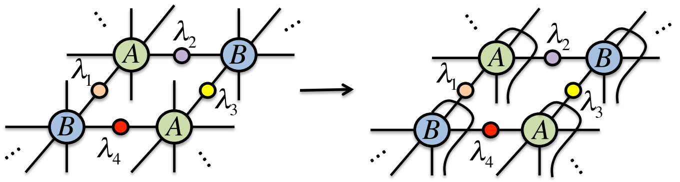

SUPPLEMENTARY FIG. 1: **From operator to vector.** PEPO on an infinite $2d$ lattice, with a 2-site unit cell, tensors A and B at sites, and $\lambda_1, \lambda_2, \lambda_3$ and λ_4 at the links, as well as its vectorization.

Supplementary Note 2: Simple update

The time evolution generated by the Liouvillian superoperator is broken via a Trotter decomposition into small two-body gates, namely

$$e^{T\mathcal{L}_\sharp} = \left(e^{\delta t \mathcal{L}_\sharp}\right)^{T/\delta t} \approx \left(\prod_{\langle i,j \rangle} e^{\delta t \mathcal{L}_\sharp^{[i,j]}}\right)^{T/\delta t} \equiv \left(\prod_{\langle i,j \rangle} g^{[i,j]}\right)^{T/\delta t} , \tag{1}$$

where we implemented for concreteness the first-order Trotter approximation, and we defined the 2-body gates $g^{[i,j]}$ acting on the different links. The action of one of these gates in a given link is accounted for by defining new approximated PEPO tensors following the scheme in Supplementary Fig.(2), called *simple update* [1–5]. This is the direct generalization of the update rule of the TEBD algorithm for $1d$ MPS [6, 7]. The update is locally optimal in $1d$, whereas only approximate in $2d$ because it does not take into account the effect of the environment of the link in the approximation of the tensors. Still, it is remarkably efficient, and produces good results for gapped phases with small correlation length.

Supplementary Note 3: Local observables

The calculation of local observables follows from an approximate contraction of the $2d$ tensor network using corner transfer matrices (CTM) [8–15]. For instance, the calculation of the (unnormalized) 1-site density matrix is done as shown in Supplementary Fig.(3). First, square-roots of the λ tensors are contracted with the tensors at every site as in Supplementary Fig.(3(a)). The partial trace is taken as in Supplementary Fig.(3(b)), which produces a tensor network as in Supplementary Fig.(3(c)). This tensor network is approximated using four CTMs C_1, C_2, C_3 and C_4, as well as four half-row/column transfer matrices Ta_u, Ta_r, Ta_d and Ta_l as in Supplementary Fig.(3(d)) – which would be eight for a 4 site unit cell –. These approximating tensors are the *effective environment* of the site where we compute the reduced density matrix.

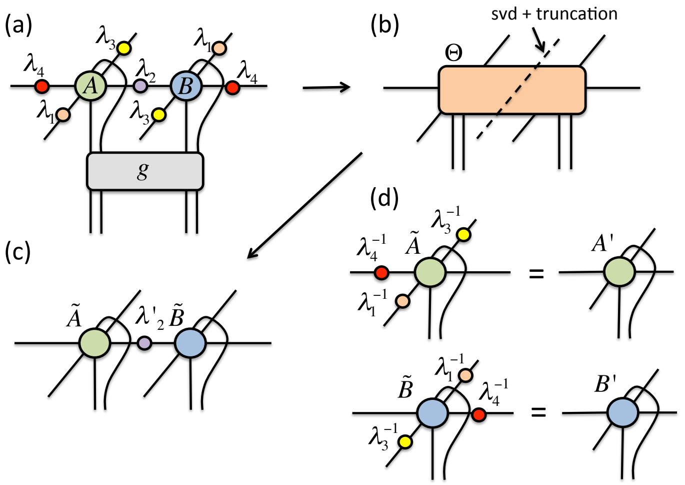

SUPPLEMENTARY FIG. 2: **Tensor update with the simple update.** A 2-body gate g acts on a given link of the PEPO as in (a), and the contraction produces tensor Θ as in (b). This tensor is broken into two pieces by a singular value decomposition (svd), and after truncation of the singular values in D (by keeping the D largest ones) it produces the structure in (c). The new tensor λ_2' corresponds to the truncated singular values. The new tensors A' and B' at the sites are computed as in (d).

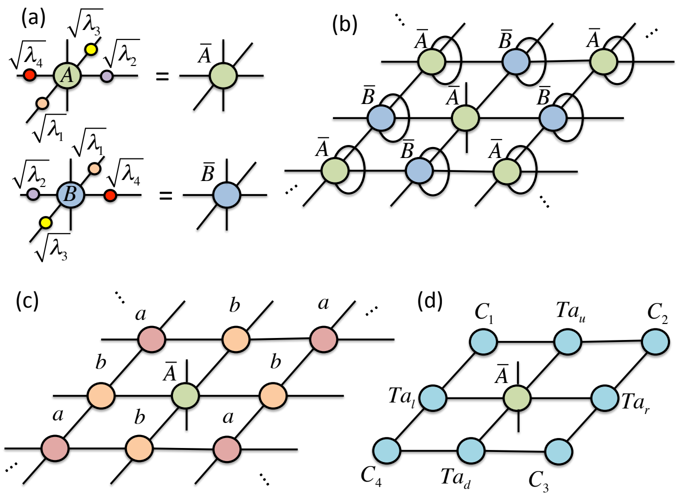

SUPPLEMENTARY FIG. 3: **Computing a 1-site reduced density matrix.** (a) Square roots of the λ tensors at the links are contracted with the tensors at the sites, in order to have a tensor network with one tensor per site; (b) partial trace over the environment of one site in the lattice; (c) tensor network obtained as a result of the partial trace; (d) approximation of the contraction in (c) in terms of CTMs and half row/column tensors.

In Supplementary Fig.(4) we provide the basic details of how the tensors for the effective environment are computed [8–15]. In particular, for a “left move”, two rows are inserted in the network and absorbed towards the left. The growth of the bond index is renormalized by an isommetry W (see Supplementary Fig.(4(c)), which can be computed according to several prescriptions [8–15]. The procedure follows by iterating directional moves along the left, right, up and down directions until convergence.

Supplementary Note 4: Operator-entanglement entropy

We define the operator-entanglement entropy as

$$S_{\rm op}(\rho) \equiv -{\rm tr}\left(\sigma_\sharp \log_2 \sigma_\sharp\right) , \tag{2}$$

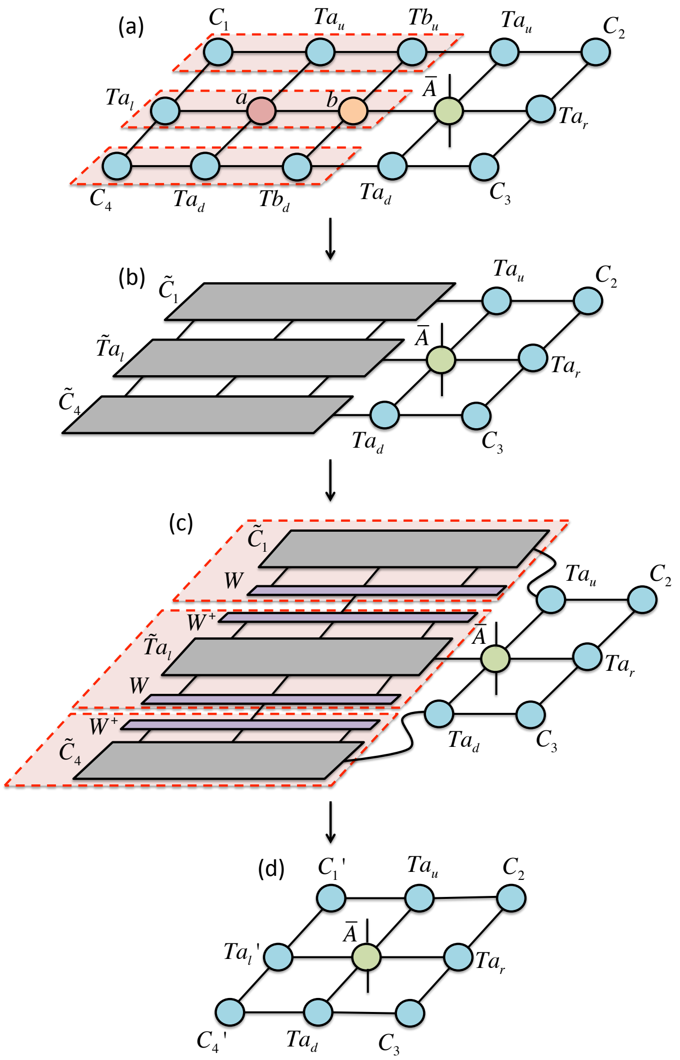

SUPPLEMENTARY FIG. 4: **Left move of the iterative procedure to compute the approximate environment.** We follow the step in Ref.[13]. (a) Two columns are inserted; (b) new exact left tensors are defined; (c) renormalization of the bond indices by an isometry W; (d) new renormalized environment tensors on the left.

with

$$\sigma_\sharp \equiv \mathrm{tr}_{\mathrm{E}} \left(|\rho\rangle_\sharp \ {}_\sharp\langle\rho| \right). \tag{3}$$

In the above equations, $|\rho\rangle_\sharp$ is the vectorized reduced density matrix, and tr_E is the partial trace over the sites for which we wish to compute the entropy. In a nutshell: this is the entanglement entropy of $|\rho\rangle_\sharp$, the vectorized density matrix, understood as a pure state. As such, this is *not* a measure of entanglement of the mixed state ρ. However, this is the relevant measure of correlations for our purposes, since it is upper-bounded directly by the bond dimension of the PEPO. Namely, if the PEPO has bond dimension D, then for a block of $L \times L$ sites one has

$$S_{\mathrm{op}}(\rho) \leq 4L \log_2 D, \tag{4}$$

which means that we can use it to quantify how large needs to be our bond dimension D for the PEPO, being this directly connected to the computational cost and the accuracy of the method [16].

In what follows we explain to procedures to compute $S_{\mathrm{op}}(\rho)$: one fully taking into account the environment of the block, and one approximate taking into account some of the properties of the simple update.

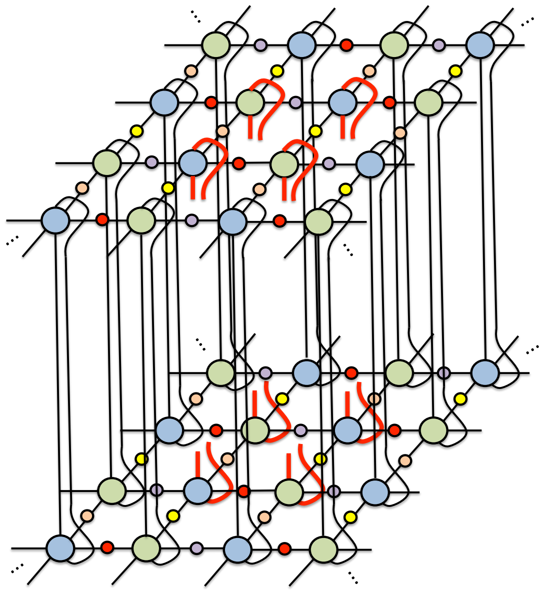

SUPPLEMENTARY FIG. 5: **Full calculation of operator entanglement entropy.** Tensor network for the operator $\sigma_\sharp$, obtained after tracing out the degrees of freedom of the environment of a 2×2 block in the vector $|\rho\rangle_\sharp$. We omit the name of the tensors for clarity of the diagram. Open indices of $\sigma_\sharp$ are shown in red.

Full calculation

The calculation taking into account the full environment follows the tensor contraction from Supplementary Fig.(5). In this case, we compute $\sigma_\sharp$ by tracing out the degrees of freedom outside the block, as shown in the figure. The corresponding contraction can be approximated in the thermodynamic limit using the CTM method explained above.

Simple calculation

For the case of a phase with small correlation length, and using the information obtained from the simple update (namely, tensors at the sites and at the links), it is indeed possible to approximate, up to a good accuracy, the tensor network in Supplementary Fig.(5) by the one in Supplementary Fig.(6). In this approximation, one does not take the surrounding environment of the block fully into account. Instead, the effect of the environment is replaced by the effect of the λ tensors surrounding the block, which amounts to a mean-field approximation of the effective environment. Moreover, one can see that in such a case the eigenvalues of $\sigma_\sharp$ can be approximated with good accuracy by the product of the squares of the surrounding λ tensors, i.e.,

$$\mathrm{eig}(\sigma_\sharp) \approx \prod_{i \in \mathrm{boundary}} \left(\lambda^{[i]}\right)^2 , \tag{5}$$

and therefore the operator entanglement entropy reads

$$S_{\mathrm{op}}(\rho) \approx \sum_{i \in \mathrm{boundary}} S_{\mathrm{op}}^{[i]} \tag{6}$$

with

$$S_{\mathrm{op}}^{[i]} \equiv - \sum_{\alpha=1}^{D} \left(\lambda_\alpha^{[i]}\right)^2 \log_2 \left(\lambda_\alpha^{[i]}\right)^2 . \tag{7}$$

This approximation works very well in gapped phases computed via the simple update, and it is the one that we used in the main text.

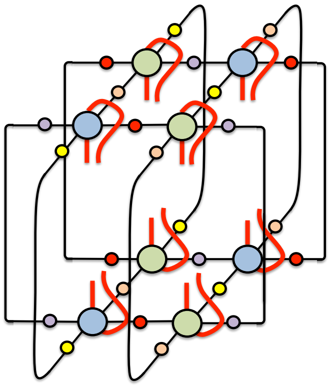

SUPPLEMENTARY FIG. 6: **Simple calculation of operator entanglement entropy.** Tensor network for the operator $\sigma_\sharp$ for a 2×2 block in the vector $|\rho\rangle_\sharp$, with a mean-field approximation of the effective environment. We omit the name of the tensors for clarity of the diagram. Open indices of $\sigma_\sharp$ are shown in red.

Supplementary References

[1] R. Orús, "A Practical Introduction to Tensor Networks: Matrix Product States and Projected Entangled Pair States", Annals of Physics **349** 117-158 (2014).

[2] J. Eisert, "Entanglement and tensor network states", Modelling and Sim. **3**, 520 (2013).

[3] N. Schuch, "Condensed Matter Applications of Entanglement Theory ", QIP, Lecture Notes of the 44th IFF Spring School (2013).

[4] J. I Cirac and F. Verstraete, "Renormalization and tensor product states in spin chains and lattices", J. Phys. A: Math. Theor. **42**, 504004 (2009)

[5] F. Verstraete, J. I. Cirac and V. Murg, "Matrix Product States, Projected Entangled Pair States, and variational renormalization group methods for quantum spin systems ", Adv. Phys. **57**, 143 (2008).

[6] G. Vidal, "Efficient classical simulation of slightly entangled quantum computations", Phys. Rev. Lett. **91**, 147902 (2003).

[7] G. Vidal, "Efficient simulation of one-dimensional quantum many-body systems", Phys. Rev. Lett. **93**, 040502 (2004).

[8] R. J. Baxter, "Corner transfer matrix", Physica A **106**, pp18-27 (1981).

[9] R. J. Baxter, "Exactly Solved Models in Statistical Mechanics", Academic Press, London, (1982).

[10] R. J. Baxter, "Dimers on a rectangular lattice", J. Math. Phys. **9**, 650 (1968).

[11] R. J. Baxter, "Variational approximations for square lattice models in statistical mechanics", J. Stat. Phys. **19** 461 (1978).

[12] T. Nishino and K. Okunishi, "Corner Transfer Matrix Renormalization Group Methods", J. Phys. Soc. Jpn. **65** pp. 891-894 (1996).

[13] T. Nishino and K. Okunishi, "Corner Transfer Matrix Algorithm for Classical Renormalization Group", J. Phys. Soc. Jp. **66**, 3040 (1997).

[14] R. Orús and G. Vidal, "Simulation of two dimensional quantum systems on an infinite lattice revisited: corner transfer matrix for tensor contraction", Phys. Rev. B **80**, 094403 (2009).

[15] R. Orús, "Exploring corner transfer matrices and corner tensors for the classical simulation of quantum lattice systems", Phys. Rev. B **85**, 205117 (2012).

[16] S. Sahin, R. Orús, K. P. Schmidt, "Entanglement Continuous Unitary Transformations", Europhysics Letters **117**, 20002 (2017).
